# Significant risk factors for malignant transformation of ovarian endometrioma during dienogest treatment: a case report and retrospective study

**DOI:** 10.1186/s13256-019-2236-z

**Published:** 2019-10-22

**Authors:** Michiko Honda, Wataru Isono, Akira Tsuchiya, Ako Saito, Hiroko Tsuchiya, Reiko Matsuyama, Akihisa Fujimoto, Osamu Nishii

**Affiliations:** Department of Obstetrics and Gynaecology, University Hospital Mizonokuchi, Teikyo University School of Medicine, Kanagawa, 5-1-1, Kawasaki, Takatsu-ku, Futago, Kanagawa 213-8507 Japan

**Keywords:** Endometrioma, Multivariate analysis, Progestin, Retrospective study, Tumorigenic transformation

## Abstract

**Background:**

To determine the prevalence of and risk factors for malignant transformation of ovarian endometrioma during dienogest therapy, which is very rare, we examined multiple cases of malignant transformation of ovarian endometrioma during dienogest therapy and performed a multivariate analysis of the records in our hospital.

**Methods:**

The medical records of 174 patients who underwent DNGT for the treatment of OMA from June 1, 2011, to May 31, 2018, were reviewed retrospectively with the approval of the Human Ethical Committee of the University of Teikyo Hospital. And we provided one representative case of MT with obtaining written informed consent. To assess the effects of six representative factors, including advanced age, parity, surgical history, and endometrial cyst characteristics (including 3 factors), on the possibility of malignant transformation, we performed a multivariate logistic regression analysis.

**Results:**

Of the 174 cases, 4 were diagnosed with malignant transformation, and these cases are reported. In the multivariate analysis, advanced age (*P* = 0.0064), nullipara (*P* = 0.0322), and enlargement (*P* = 0.0079) showed significant differences for malignant transformation occurrence. All 4 malignant transformation cases were among the 19 patients who had all of these 3 factors.

**Conclusions:**

For a more accurate determination of the treatment approach, a larger sample size will be needed to determine the risk factors for malignant transformation during dienogest therapy.

## Background

Endometriosis is among the most common gynecological diseases and is known to affect 2–10% of women of reproductive age [1, 2]. This disease most commonly affects the ovaries, forming a cystic mass called an ovarian endometrioma (OMA) [3], and is diagnosed by an outpatient ultrasound examination. In many cases, magnetic resonance imaging (MRI) is needed for a definitive diagnosis [4]. Because endometriosis is frequently associated with infertility and pelvic pain [5], laparoscopic surgery or drug treatment is often performed for symptom relief [6]. The use of dienogest therapy (DNGT), a drug-based treatment, has recently expanded because DNGT has a curative effect in terms of pelvic pain [7] and is expected to reduce the size of an OMA [8]. This therapy can also reduce postoperative lesion recurrence and extend the pain-free period [9]. Thus, dienogest is a new and effective drug. However, several adverse effects, including uterine bleeding, have been reported [10]. Among these side effects, some cases of malignant transformation (MT) during DNGT have been reported [11], although MT during DNGT is very rare. However, a detailed analysis of this risk factor has not been performed. For this reason, in addition to the present report of several cases of MT during DNGT in our hospital, we have performed a multivariate analysis of the risk factors for MT during DNGT.

Recent studies have indicated that endometrioid carcinoma and clear cell carcinoma are associated with OMA [12]. Ovarian endometrioma is a subtype of endometriosis, affecting 17–44% of women with endometriosis [13]. In most cases, this disease is diagnosed as endometrioma by an outpatient ultrasound examination [4], and laparoscopic surgeries are often performed to treat pelvic pain and subfertility [6]. In a previous report, these cancers reportedly developed in 0.72% of women with a previous diagnosis of OMA who had been managed expectantly [14]. Conversely, some reports showed that a certain ratio of patients with ovarian cancers have a history of endometriosis [15, 16]. However, no significant relationship between previous treatments with hormone drugs (or surgery) and the risk of MT has been validated [17, 18]. Therefore, our analysis may provide new insight into preventing future cases of MT of OMA.

## Methods

### Data collection

This study was reviewed and approved by the Human Ethics Committee of the University of Teikyo Hospital (trial registration number 18-233). The de-identified medical records of 185 female patients who received dienogest for the treatment of OMA between June 1, 2011, and May 31, 2018, were reviewed retrospectively with the approval of the Human Ethical Committee of the University of Teikyo Hospital. And we provided one representative case of MT with obtaining written informed consent. In this study, we included patients with and without surgery for OMA. Of the 185 patients, 11 patients were excluded because of insufficient data. Among the 174 remaining cases were 4 cases with MT of OMA, and the following data were collected: (1) the size of the OMA before and after starting DNGT (when the patients had bilateral OMA, we adopted the larger OMA from the right or left side), (2) the patient’s parity (nulliparous or multiparous patient), (3) the patient’s age at the time of starting DNGT, (4) the location of the endometrial cyst before starting DNGT (unilateral or bilateral), and (5) whether surgery was performed. In this study, we focused mostly on the change in the cyst size. In addition, 174 cases were classified into the following three groups: (1) “reduction,” defined as cases in which the cyst size showed a decline of 30% or more; (2) “enlargement,” defined as cases in which the cyst size showed an increase of 30% or more or in which a new cyst appeared after surgery; and (3) “no change,” which included the remaining cases.

Usually, when treating patients with endometriosis, we use the revised American Society for Reproductive Medicine (rASRM) score to classify the severity [19]. However, due to insufficient data, we could not include the rASRM score in the multivariate analysis. Because not all patients underwent surgery, we were able to obtain data regarding the rASRM score for only 73 cases (42%; *n* = 73 of 174). When an ovarian endometrioma is detected, the severity of endometriosis is regarded as moderate or severe, meaning that the severity of endometriosis is at least stage 3 among four stages. Therefore, all patients in this analysis had a certain level of endometriosis severity. Additionally, we did not analyze the treatment period of dienogest, because DNGT was generally used for the long term and was continued until menopause in some cases [20].

### Statistical analysis

We performed a multivariate logistic regression analysis to assess the influence of the following six factors on the possibility of MT: (1) “advanced age,” defined as patients who started DNGT at 41 years of age or older; (2) nullipara; (3) “large cyst,” defined as patients whose cyst sizes were 8 cm or larger at the start of DNGT; (4) “surgical history,” defined as patients who underwent surgery for OMA; (5) bilateral cysts; and (6) enlargement. These statistical analyses were performed using JMP version 12 for Windows (SAS Institute, Inc., Tokyo, Japan). The data are presented as the means ± standard deviations. A *P* value less than 0.05 was considered statistically significant.

## Case presentation

**Fig. 1:**
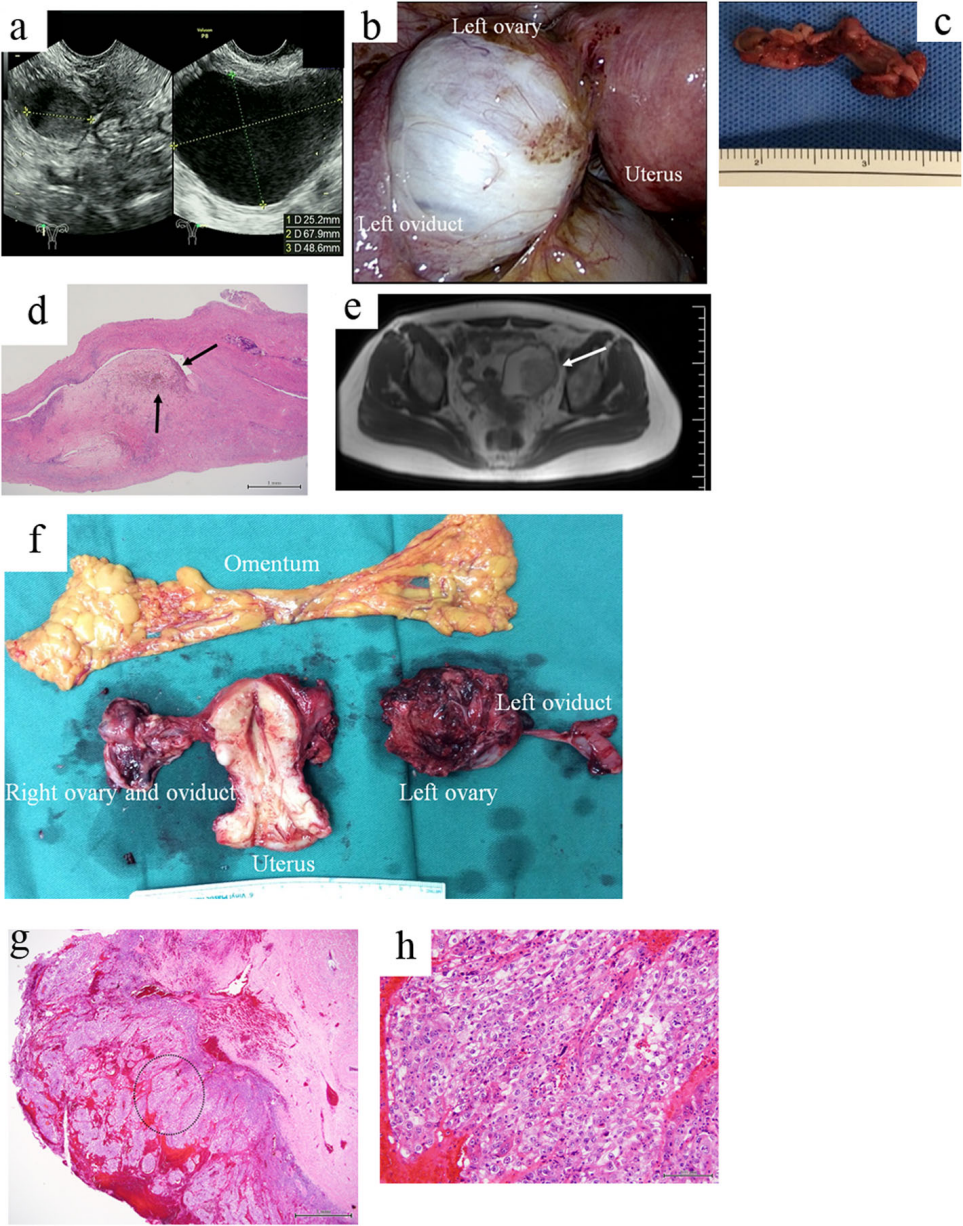
Images and findings of reported case of malignant transformation. **a** The sizes of the left and right cysts were 7 cm and 3 cm in diameter, respectively, and the cysts were diagnosed by transvaginal ultrasound examination 1 day before surgery. **b** Intraoperative image of the left ovarian endometrioma (OMA). The ovarian cyst contains chocolate-like fluid. **c** Gross appearance of the left ovary at laparoscopic cystectomy (LC). The ovarian cyst does not contain a solid component. **d** Pathological findings of the left OMA (H&E staining, conventional light microscopy at a magnification of ×20). Abundant hemosiderin-laden macrophages were present in the cyst wall, as indicated by *arrows*. **e** T2-weighted magnetic resonance image at diagnosis of malignant transformation. There was a cystic left ovarian mass (7 cm in diameter) with a solid component (*arrow*). One scale = 1 cm. We could not obtain a magnetic resonance image before LC, because the patient had undergone a magnetic resonance imaging examination before visiting our hospital. **f** Gross appearance of left ovarian carcinoma and other organs removed at surgery for ovarian cancer. The ovarian cyst contains a large solid component with chocolate-like fluid. **g**, **h** Pathological findings of the left ovarian clear cell carcinoma, as indicated by a *circle* (H&E stain, original magnification in **g** ×20 and **h** ×200). Tumor cells have eosinophilic cytoplasm and hyalinized stroma

A 43-year-old Japanese woman, gravida 0, para 0, was referred to our hospital because of aggravated dysmenorrhea and hypermenorrhea. An ovarian cyst had been noted in this patient 3 years before her visit to our hospital, and she had dysmenorrhea beginning at approximately 30 years of age. MRI that was performed before her visit to our hospital suggested a left endometrioma that was 7 cm in diameter and a right endometrioma that was 3 cm in diameter, with no solid component in either endometriotic cyst. Bilateral laparoscopic cystectomy (LC) was performed with the patient under general anesthesia, and the diameters of the right and left endometriomas were 3 cm and 7 cm, respectively (Fig. 1a and b). After both ovaries were subjected to a surgical procedure, including complete Douglas pouch obliteration, to release them from their severe pelvic adhesion, the left endometrial cyst was completely excised, and the right endometrial cyst was ablated. The rASRM score was 81 points, and this case was diagnosed as stage IV endometriosis. The ovarian cyst did not contain a solid component (Fig. 1c), and pathological examination showed a left endometriotic cyst and no malignancy (Fig. 1d). After surgery, DNGT was chosen for the prevention of recurrence, and the patient was monitored by transvaginal ultrasound every 6 months.

At 1 year and 1 month after surgery (that is, 1 year after the start of the administration of DNGT), when the patient was 44 years old, a recurrent left-sided OMA that was 6 cm in diameter with a solid component inside the left ovarian tumor was detected by outpatient transvaginal ultrasound examination. Contrast-enhanced MRI showed that in addition to a 3-cm right endometrioma, there was a 7-cm left endometrioma with a solid component inside the cavity in which MT was strongly suspected (Fig. 1e). In addition, contrast-enhanced computed tomography exhibited numerous para-aortic lymph node enlargements, including a maximum-sized node that was 10 mm in diameter. The serum cancer antigen 125 level, which was 51.4 U/ml at the time of the first visit to our hospital, was increased to 515 U/ml. Soon after the imaging diagnosis was performed, complete abdominal surgery, including removal of the uterus, bilateral ovaries, omentum, pelvic lymph nodes, and para-aortic lymph nodes, was performed. The final diagnosis was clear cell carcinoma of the left ovary (Fig. 1f, g, h), International Federation of Gynecology and Obstetrics (FIGO) stage 1C1, pT1C1N0M0. After the necessity of chemotherapy was explained to her, she received chemotherapy of 175 mg/m^2^ paclitaxel and carboplatin (TC; area under the curve, 6 mg/ml/min) for six cycles and was without recurrence during follow-up.

## Results

### Patient characteristics

In the 174 patients with DNGT, the average endometrioma size before the start of therapy, patient age, and observable treatment period were 4.9 ± 2.2 cm, 39.7 ± 5.9 years, and 27.3 ± 21.6 months, respectively. There were 70 patients with bilateral OMAs, 51 patients with right-sided OMAs, and 53 patients with left-sided OMAs. We were able to divide the 174 patients into 57 “reduction” cases, 70 “enlargement” cases, and 47 “no change” cases. All four cases of MT during DNGT were included in the “enlargement” group. Therefore, when we compared the change in cyst size, 57 “reduction” cases and 47 “no change” cases were treated as similar cases in the subsequent multivariate analysis.

### Summary of four malignant transformation cases

Table 1 lists the characteristics of the four patients who developed ovarian cancer during DNGT. In this study, we included two patients who were referred to our hospital because the possibility of ovarian cancer during DNGT had been noted in other hospitals. Therefore, 2 of 172 patients who underwent DNGT were diagnosed with MT in our hospital, and the probability reached 1.2%. Of four patients, two patients began DNGT after LC. All four patients were nulliparous. The ages at the time of DNGT ranged from 42 to 44, and the ages at the time of diagnosis of MT ranged from 42 to 46. The duration of DNGT ranged from 9 to 33 months. Two patients had bilateral OMAs, and two patients had a left OMA. Of four cases, three cases of ovarian cancer developed from a left-sided OMA, and one case of ovarian cancer developed from a right-sided OMA after experiencing recurrence or enlargement of the OMA. As shown in Table 1, the ovarian cancers for all four cases were detected in the early stage and were FIGO stage 1C1 (three cases) or 1C3. Complete surgery, including the removal of the uterus, bilateral ovaries, omentum, pelvic lymph nodes, and para-aortic lymph nodes, was performed in two cases (cases 1 and 3). For the remaining two cases, we performed total hysterectomy and bilateral salpingo-oophorectomy (case 4) or left-sided salpingo-oophorectomy (case 2). For case 2, we performed interval debulking surgery, including the removal of the uterus, right ovary, omentum, pelvic lymph nodes, and para-aortic lymph nodes, after the completion of three cycles of TC because the pathological diagnosis was clear cell carcinoma. While performing interval debulking surgery, we detected cancer recurrence in the abdominal cavity and diagnosed the cancer as FIGO stage 3C. After some treatments, including irinotecan, bevacizumab, and oral etoposide, the patient died of extensive metastasis and cancerous peritonitis.

**Table 1:** Summary of four cases

	Case 1	Case 2	Case 3	Case 4
Patient’s parity	Nullipara	Nullipara	Nullipara	Nullipara
Age at start of DNGT	43 years	44 years	42 years	44 years
Age at MT	44 years	46 years	42 years	46 years
Cyst size before DNGT	7 cm (left), 3 cm (right)	8 cm (left)	4 cm (left)	3 cm (left)
Cyst size at MT	7 cm (left), 3 cm (right)	5 cm (left)	7 cm (left), 2 cm (right)	3 cm (left), 2 cm (right)
Duration of DNGT	14 months	31 months	9 months	33 months
Operation for ovarian carcinoma	TAH, BSO, OM, PLA, PALA	LSO	TAH, BSO, OM, PLA, PALA	TAH, BSO, OM
Pathology	Clear cell carcinoma	Clear cell carcinoma	Clear cell carcinoma	Clear cell carcinoma
Origin of carcinoma	Left ovary	Left ovary	Left ovary	Right ovary
FIGO stage	1C1	1C3	1C1	1C1
Surgical history (age)	Bilateral LC (43 years)	Left-side LC (40 years)	No	No
Chemotherapy	TC for 6 cycles	TC for 3 cycles	TC for 6 cycles	TC for 6 cycles
Serum CA 125 at MT	515 U/ml	78.5 U/ml	42.4 U/ml	46.3 U/ml

*Abbreviations*: *BSO* Bilateral salpingo-oophorectomy, CA 125 Cancer antigen 125, *DNGT* Dienogest therapy, *FIGO* International Federation of Gynecology and Obstetrics, *LC* Laparoscopic cystectomy, *LSO* Left salpingo-oophorectomy, *MT* Malignant transformation, *OM* Omentectomy, *PALA* Para-aortic lymphadenectomy, *PLA* Pelvic lymphadenectomy, *RSO* Right salpingo-oophorectomy, *TAH* Total abdominal hysterectomy, *TC* Paclitaxel and carboplatin

### Influential factors in malignant transformation

To determine significant factors associated with the possibility of MT, we compared the following six factors using multivariate logistic regression models (Table 2): (1) advanced age (*n* = 45), (2) nullipara (*n* = 113), (3) large cyst (*n* = 18), (4) surgical history (*n* = 81), (5) bilateral cysts (*n* = 70), and (6) enlargement (*n* = 70). This analysis revealed that advanced age (*P* = 0.0064), nullipara (*P* = 0.0322), and enlargement (*P* = 0.0079) were significant factors for MT. In particular, all four MT cases were included among 19 patients who had the aforementioned three factors. Therefore, if the patients had these three factors, the possibility of MT reached more than 20% when choosing DNGT.

**Table 2:** Identification of influential factors for malignant transformation

	Number	Probability	*P* value
Advanced age	94	4.3%	0.0064
Nullipara	113	3.5%	0.0322
Large cyst	18	5.6%	0.1964
Bilateral cysts	70	4.3%	0.3231
Surgical history	81	2.5%	0.1814
Enlargement	70	5.7%	0.0079
Total	174	2.3%	

A multivariate analysis of 174 patients with DNGT was performed to examine the influence of six representative factors that could be collected before starting DNGT, as follows: (1) “advanced age,” defined as patients who started DNGT at 41 years of age or older; (2) nullipara; (3) “large cyst,” defined as patients whose cyst sizes were 8 cm or larger at the start of DNGT; (4) “surgical history,” defined as patients who underwent surgery for the OMA; (5) bilateral cysts; and (6) enlargement. The number of patients with each factor, the probability of MT, and the *P* values are shown. “Advanced age,” “nullipara,” and “enlargement” were identified as significant risk factors for MT. “Large cyst,” “bilateral cysts,” and “surgical history” were not significant risk factors

Abbreviations: *DNGT* dienogest therapy, *MT* malignant transformation, *OMA* ovarian endometrioma

## Discussion

Currently, DNGT is being used for patients with symptomatic endometriosis with or without surgery [21]. This progestin agent is reported to decrease the risk of recurrence by inhibiting the growth of endometriotic tissue [21, 22]. This mechanism can also theoretically prevent MT [23], and reported cases of MT are very rare [24]. However, in our hospital, we had four cases in which ovarian carcinoma was diagnosed during DNGT for OMA. MT in two of the four cases was detected entirely in our hospital, and the probability of MT after starting DNGT was 1.2% (*n* = 2 of 172). This probability was similar to the ability of endometriosis to transform into malignancy, which has been reported to be approximately 1% of OMAs in previous studies [14]. In this study, we extracted the details of the patients’ clinical histories. Because all four patients were over 40 years old and nulliparous, we hypothesized that there were several risk factors, including the patient’s age and parity, for using DNGT. To verify the significant influence of risk factors on MT, a multivariate analysis of six factors extracted before starting DNGT was performed (Table 2). Of the six factors, advanced age, nullipara and increased size and recurrence showed significant increases in the probability of MT. In contrast to a previous study in which the association between spontaneous pregnancy and the disease progression of endometriosis was unclear [25], these results were roughly consistent with those of past reports [26, 27]. In particular, 19 patients who had all three factors showed a probability of over 20% for the occurrence of ovarian carcinoma (21.1%, *n* = 4 of 19). Because the sample number was very small, the result for this extracted high-risk group might be coincidental. Therefore, a larger sample size will be needed to determine the risk factors for MT. However, in contrast to previous studies, large cyst [26–28] and surgical history [29] did not show a significantly higher probability of MT. “Bilateral cysts” were also not significant. These multivariate analysis results presented the possibility that the characteristics of the patient have a stronger influence on MT than the characteristics and treatment approach of the OMA itself. Because two cases among the four cases of MT exhibited recurrences of OMA after LC and because the other two cases exhibited cyst size enlargement after starting DNGT, careful observation of the cyst size is important, regardless of the use of surgical treatment. However, similar to previous reports [11], in our hospital, we could not prevent the two cases of MT (Table 1, case 1 and case 2) by follow-up ultrasound examination at an interval of 6 months.

## Conclusions

Although DNGT seems to be effective in controlling OMA, careful observation of the cyst size is needed. Because the probability for ovarian carcinoma occurrence could exceed 20% in some cases, particularly for nulliparous women of advanced age, a more individualized choice of therapy might be needed. For a more accurate determination of the treatment approach, a larger sample size will be needed to determine the risk factors for MT.

## Data Availability

The authors agree to make all data of this study freely available.

## Abbreviations

DNGT: Dienogest therapy
FIGO: International Federation of Gynecology and Obstetrics
LC: Laparoscopic cystectomy
MRI: Magnetic resonance imaging
MT: Malignant transformation
OMA: Ovarian endometrioma
rASRM: Revised American Society for Reproductive Medicine score
TC: Paclitaxel and carboplatin
